# Gender disparities in the association between epicardial adipose tissue volume and coronary atherosclerosis: A 3-dimensional cardiac computed tomography imaging study in Japanese subjects

**DOI:** 10.1186/1475-2840-11-106

**Published:** 2012-09-10

**Authors:** Munkhbaatar Dagvasumberel, Michio Shimabukuro, Takeshi Nishiuchi, Junji Ueno, Shoichiro Takao, Daiju Fukuda, Yoichiro Hirata, Hirotsugu Kurobe, Takeshi Soeki, Takashi Iwase, Kenya Kusunose, Toshiyuki Niki, Koji Yamaguchi, Yoshio Taketani, Shusuke Yagi, Noriko Tomita, Hirotsugu Yamada, Tetsuzo Wakatsuki, Masafumi Harada, Tetsuya Kitagawa, Masataka Sata

**Affiliations:** 1 Department of Cardiovascular Medicine, The University of Tokushima Graduate School of Health Biosciences, Tokushima, Japan; 2 Department of Cardio-Diabetes Medicine, The University of Tokushima Graduate School of Health Biosciences, Tokushima, Japan; 3 Department of Radiologic Science and Technology, The University of Tokushima Graduate School of Health Biosciences, Tokushima, Japan; 4 Department of Cardiovascular Surgery, The University of Tokushima Graduate School of Health Biosciences, Tokushima, Japan; 5 Department of Cardiovascular Medicine, Kawashima Hospital, Tokushima, Japan; 6 Department of Cardiovascular Medicine and Department of Cardio-Diabetes Medicine, The University of Tokushima Graduate School of Health Biosciences, 3-18-15 Kuramoto, Tokushima, 770-8503, Japan

**Keywords:** Atherosclerosis, Gender difference, Epicardial adipose tissue, Obesity

## Abstract

**Background:**

Growing evidence suggests that epicardial adipose tissue (EAT) may contribute to the development of coronary artery disease (CAD). In this study, we explored gender disparities in EAT volume (EATV) and its impact on coronary atherosclerosis.

**Methods:**

The study population consisted of 90 consecutive subjects (age: 63 ± 12 years; men: 47, women: 43) who underwent 256-slice multi-detector computed tomography (MDCT) coronary angiography. EATV was measured as the sum of cross-sectional epicardial fat area on CT images, from the lower surface of the left pulmonary artery origin to the apex. Subjects were segregated into the CAD group (coronary luminal narrowing > 50%) and non-CAD group.

**Results:**

EATV/body surface area (BSA) was higher among men in the CAD group than in the non-CAD group (62 ± 13 vs. 33 ± 10 cm^3^/m^2^, p < 0.0001), but did not differ significantly among women in the 2 groups (49 ± 18 vs. 42 ± 9 cm^3^/m^2^, not significant). Multivariate logistic analysis showed that EATV/BSA was the single predictor for >50% coronary luminal narrowing in men (p < 0.0001). Predictors excluded were age, body mass index, hypertension, diabetes mellitus, and hyperlipidemia.

**Conclusions:**

Increased EATV is strongly associated with coronary atherosclerosis in men.

## Introduction

Epicardial adipose tissue (EAT) is the visceral fat located between the outer layer of the myocardium and the visceral pericardium [1-4]. EAT volume (EATV) is correlated with various cardiovascular risk factors, independent of abdominal visceral adiposity, body mass index (BMI), hypertension, and diabetes mellitus [5-7]. Two population-based studies, the Multi-Ethnic Study of Atherosclerosis and the Framingham Heart Study, showed that EATV is an independent risk predictor for cardiovascular disease [5,8,9]. EAT is shown to be metabolically active and the source of pro-atherogenic mediators and adipocytokines [1-5]. Because EAT and the myocardium are located close anatomically, it is predicted that cytokines/adipocytokines produced by infiltrated macrophages or by adipocytes could locally modulate myocardial function or contribute to the pathogenesis of coronary atherosclerosis [1-5]. Recently, we [10,11] and others [12] showed that proinflammatory cytokines and adipocytokines are expressed and secreted at a higher level in the adipose tissue of individuals with coronary artery disease (CAD) than in individuals without CAD.

Abdominal fat distribution is dissimilar between men and women: Visceral fat obesity is the dominant form in men, while subcutaneous fat obesity is the dominant form in women [13,14]. However, gender differences in EATV distribution and the influence of EAT on coronary atherosclerosis has never been considered. In this study, we evaluated gender disparities in EATV and its impact on coronary atherosclerosis by using 256-slice multi-detector computed tomography (MDCT).

### Subjects and methods

#### Subjects

We recruited 119 consecutive subjects who underwent 256-slice MDCT coronary angiography between October 2009 and April 2011 at the Kawashima Hospital, Tokushima, Japan. The subjects underwent MDCT if they had atherosclerotic risk factors such as age ≥65 years [15], hypertension, smoking, diabetes mellitus, or dyslipidemia or symptoms suggestive of angina pectoris. According to the 2010 Appropriate Use Criteria for Cardiac Computed Tomography [16] guidelines, cardiac CT is not necessarily recommended for asymptomatic individuals with low-to-moderate CAD risk. However, the prevalence of CAD was not negligible even in asymptomatic subgroups [17]; hence, we employed MDCT in subjects with moderate-to-high CAD risk after they were informed of the radiation-exposure related risk, and they provided written informed consent. Coronary CT angiography was performed using a 256-slice scanner (Brilliance iCT, Philips Healthcare, Amsterdam, Netherlands), and the diagnostic accuracy of the coronary CT angiography for coronary luminal narrowing was validated by routine invasive coronary angiography. Exclusion criteria included a history of cardiac surgery, iodine-based contrast allergy, or renal failure (creatinine, >1.5 mg/dL). Hypertension was defined as a systolic blood pressure of ≥140 mm Hg and/or diastolic blood pressure of ≥90 mm Hg or as the current use of antihypertensive treatment. Diabetes was defined as fasting plasma glucose of ≥6.99 mmol/L (126 mg/dL) or the current use of hypoglycemic treatment. Hyperlipidemia was defined as fasting serum LDL-cholesterol of ≥3.62 mmol/L (140 mg/dL), HDL-cholesterol of <1.03 mmol/L (40 mg/dL), triglyceride of ≥1.58 mmol/L (140 mg/dL), and/or the current use of antihyperlipidemic treatment. The subjects were segregated into 2 groups: CAD group (presence of plaques resulting in >50% luminal narrowing in the major coronary arteries) and non-CAD group (no plaque or plaques resulting in ≤50% luminal narrowing). All subjects in the CAD and non-CAD groups were further segregated into younger (age, <65 years) and older subgroup (age, ≥65 years).

### Multi-detector CT scan protocol

The Cardiac MDCT acquisition was performed with retrospective ECG-gated cardiac imaging. The MDCT scan was performed using the following parameters: detector collimation of 2 × 128 × 0.625 mm, creating 256 overlapping slices of 0.625-mm thickness via a dynamic z-flying focal spot, gantry rotation time of 0.27 s, and tube voltage 120 kVp. A current of 800–1050 mA (depending on patient habitus) was used for helical acquisitions and a current of 200 mA for axial acquisitions. Computed tomography dose index volume (CTDIV) was calculated as 74.1 ± 12.6 mGy (n = 50). The raw scan data were reconstructed with 75% of RR wave or particular optimal phase. A bolus dose of the contrast medium (Iohexol [Omnipaque; Daiichi-Sankyo Pharmaceutical, Tokyo, Japan], containing 350 mg iodine/mL) was injected at 0.7 mL/kg body weight within 9 s. Nitroglycerin (0.3 mg) was administered to all subjects immediately before CT imaging, and an oral β blocker (metoprolol, 60 or 120 mg) was administered 1 h before CT imaging to render heart rates <65 beats/min, if required.

### Analysis of EATV

**Figure 1:**
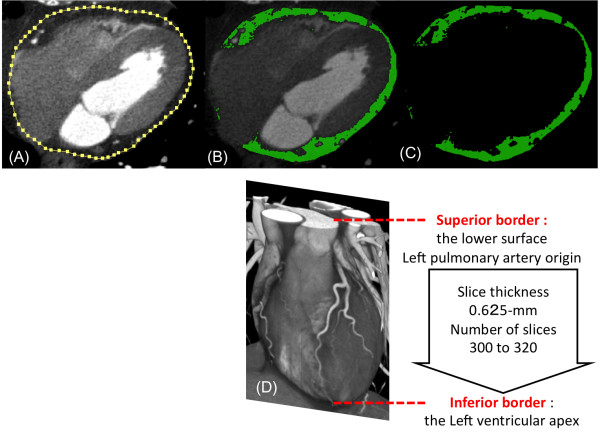
**Total epicardial adipose tissue volume (EATV) measurements on 256-slice MDCT.** (**A**)–(**C**): Axial images. A region of interest (ROI) was manually placed along the visceral pericardium (yellow line) (A) and EAT was extracted on an axial image (green) (**B, C**). (**D**) 3D image: EAT area was measured on each axial image from the lower surface of left pulmonary artery origin to the left ventricular apex and total EAT volume was obtained from multiplying EAT area and the slice thickness.

We performed volumetric quantification of EAT measured by the 256-slice MDCT, as described with some modifications [18-22] (Figure 1). Quantification of total EAT area (cm^2^) was performed at a workstation (Real-INTAGE, Kubota, Japan) with dedicated software. Volumetric measurements were performed on axial views of 0.625-mm slice thickness and number of slices ranging between 300 and 320. The superior border of the EATV measurements was the lower surface of the left pulmonary artery origin, while the inferior border was the left ventricular apex. The EAT area around the proximal, middle, and distal segment of the major coronary arteries was included in the volumetric measurements. The EAT area was calculated by tracing a region of interest (ROI), which included the heart and EAT. The ROI was manually placed outside the line of the visceral pericardium on a cross-sectional axial image (yellow line, Figure 1). The area outside the traced pericardium was excluded. A density range of -600 to -20 Hounsfield Units was used to isolate the adipose tissue. The EAT area of each slice was then summed and multiplied by the slice thickness and number of slices to determine the total EATV (cm^3^).

### Assessment of coronary atherosclerosis

The presence of coronary atherosclerosis was estimated by CT angiographical data using a 256-slice Brilliance iCT (Philips Medical Systems). Coronary arteries were assessed for luminal narrowing by using 3D visualization tools after the axial images were reviewed for determination of anatomy, quality of the study, and appearance of the vessels. The coronary artery tree was segmented according to the modified American Heart Association classification [23]. Coronary vessel and diameter were assessed on 2D multiplanar reconstruction (MPR) and 2D thin slab maximum intensity projection (MIP) images. A 3D volume rendered (VR) image was used to display long segments of the vessels and their branches. To compare the possible gender differences in the calcified lesion, we compared the Agatston scores between the men and women in CAD and non-CAD [24].

### Statistical analysis

Values are expressed as mean ± SD unless otherwise indicated. For comparison of the mean in the 2 groups, we used unpaired *t*-test when samples were normally distributed and non-parametric Mann-Whitney's *U* test when samples were not normally distributed. *χ*^2^ test was used to examine differences with categorical variables. Multigroup comparisons of variables were performed using one-way ANOVA followed by Tukey-Kramer HSD (honestly significant difference) test. Multiple logistic regression analysis was performed to adjust confounding factors. Variables were treated as continuous: one with a risk as 1 and one with no risk, i.e., as 0. We investigated the independent variables for detecting coronary artery luminal narrowing (>50%) by using unadjusted (univariate) and adjusted style (multivariate) for age, BMI, and other established risk factors (hypertension, hyperlipidemia, and diabetes mellitus). All analyses were performed using Jump version 9.0.2 software (SAS Institute Inc., Cary, NC). P values less than 0.05 were considered significant.

### Results

#### General characteristics

Of the 119 subjects who underwent cardiac MDCT, 29 were excluded from analysis. Among the men, 11 were excluded because of differences in slice levels and 2 because of insufficient image quality; among the women, 11 were excluded because of differences in slice levels and 5 because of insufficient image quality. A total of 90 subjects (men: n = 47; age = 63 ± 12 years; women: n = 43, age = 64 ± 12 years) were analyzed (Table 1). By using MDCT, 22 men and 16 women were segregated into the CAD group (>50% luminal narrowing), while 25 men and 27 women were segregated into the non-CAD group. Among the 47 men, 36 (77%) had normal weight (BMI < 25 kg/m^2^), 8 (17%) were overweight (BMI 25–30 kg/m^2^), and 3 (6%) were obese (BMI > 30 kg/m^2^). Among the 43 women, 33 (77%) had normal weight (BMI < 25 kg/m^2^), 7 (16%) were overweight (BMI 25–30 kg/m^2^), and 3 (7%) were obese (BMI > 30 kg/m^2^). When the non-CAD and CAD groups were combined, we found that the EATV was higher in men than in women (80 ± 33 vs. 65 ± 21 cm^3^; p = 0.0089), but the mean EATV/height and EATV/BSA were comparable (Table 1).

**Table 1:** Characteristics of the study population

	**Men**				**Women**				
	**All**	**non-CAD**	**CAD**	**P1**	**All**	**P2**	**non-CAD**	**CAD**	**P1**
Patient, n	47	25	22		43		27	16	
Age (years)	61 ± 13	56 ± 13	67 ± 9	0.0025	66 ± 12	ns	65 ± 10	65 ± 13	ns
Body weight (kg)	65.9 ± 10.9	66.4 ± 12.3	65.3 ± 9.2	ns	53.4 ± 1.5	0.001	54.8 ± 8.5	51.0 ± 9.5	ns
Body mass index (kg/m^2^)	24.0 ± 3.5	24.2 ± 4.4	23.9 ± 2.0	ns	23.3 ± 3.5	ns	23.9 ± 3.0	22.3 ± 4.1	ns
Systolic blood pressure (mmHg)	134 ± 2	136 ± 17	131 ± 12	ns	135 ± 3	ns	132 ± 14	140 ± 24	ns
Diastolic blood pressure (mmHg)	76 ± 1	75 ± 7	77 ± 9	ns	77 ± 6	ns	77 ± 6	77 ± 8	ns
LDL-cholesterol (mmol/L)	2.54 ± 0.49	2.47 ± 0.46	2.52 ± 0.61	ns	3.00 ± 0.60	0.01	3.11 ± 0.49	2.90 ± 0.28	ns
HDL-cholesterol (mmol/L)	1.09 ± 0.28	1.09 ± 0.18	0.97 ± 0.15	ns	1.63 ± 0.54	<0.0001	1.68 ± 0.52	1.89 ± 0.34	ns
Triglyceride (mmol/L)	1.47 ± 0.46	2.07 ± 1.84	1.82 ± 0.67	ns	1.50 ± 0.82	ns	1.39 ± 0.04	1.25 ± 0.44	ns
Glucose (mmol/L)		7.55 ± 2.72	6.83 ± 1.61	ns			6.27 ± 1.67	9.71 ± 7.99	ns
HbA_1c_ (NGSP %)	6.85 ± 1.72	6.85 ± 0.57	6.84 ± 0.50	ns	6.15 ± 0.98	ns	6.39 ± 0.35	6.35 ± 0.75	ns
Hypertension (%)	71	63	81	ns	58	ns	58	60	ns
Diabetes mellitus (%)	40	33	48	ns	17	0.020	12	30	ns
Hyperlipidemia (%)	83	78	83	ns	79	ns	79	80	ns
EATV (cm^3^)	80 ± 33	57 ± 21	106 ± 23	<0.0001	65 ± 21	0.009	62 ± 13	68 ± 29	ns
EATV/height (cm^3^/m^2^)	47 ± 19	96 ± 37	176 ± 40	<0.0001	45 ± 14	ns	94 ± 6	105 ± 45	ns
EATV/BSA (cm^3^/m^2^)	48 ± 20	33 ± 10	62 ± 13	<0.0001	42 ± 14	ns	42 ± 9	49 ± 18	ns
Agatston score	502 ± 926	582 ± 400	400 ± 637	ns	277 ± 589	ns	255 ± 580	401 ± 617	ns

Values are means ± SD. P1:p values vs Non-CAD, P2:p values vs Men. ns: not significant. *CAD*: coronary artery disease; *LDL*: low density lipoprotein; *HDL*: High density lipoprotein; *HbA*_*1c*_ : Glycosylated hemoglobin, *NGSP*: National Glycohemoglobin Standardization Program, *EATV*: epicardial adipose tissue volume, *BSA*: body surface area.

### Distribution of BMI and EATV/BSA in non-CAD and CAD subjects

**Figure 2:**
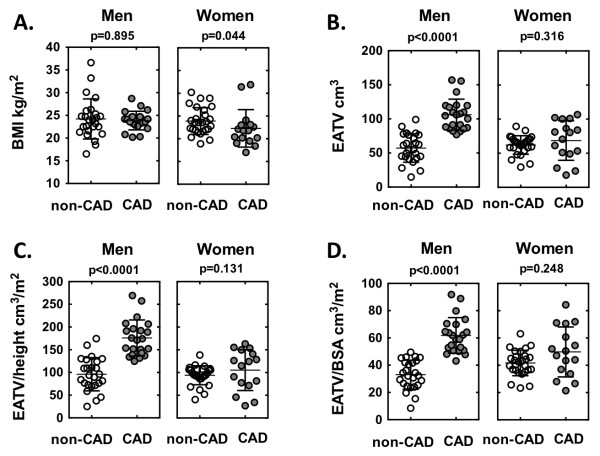
**Comparison of BMI (A), EATV (B), EATV/height (C), and EATV/BSA (D) in non-CAD (○) and CAD (●) subjects.** BMI, body mass index; EATV, epicardial adipose tissue volume; BSA, body surface area. Coronary artery disease (CAD) was defined if one has plaque lesion(s) resulting in >50% luminal narrowing.

EATV, EATV/height, and EATV/BSA were considerably higher among men in the CAD group than in those in the non-CAD group (Figure 2B-D) but not significantly different among women in the non-CAD and CAD groups (Figure 2B-D). BMI was not different in both groups (Figure 2A). Because age was higher in the CAD group than in the non-CAD group (Table 1), we tried to minimize the confounding effects of age by comparing patients in the younger (<65 years) and older (≥65 years) subgroups. EATV, EATV/height, and EATV/BSA were higher among men in the CAD group than in the non-CAD group both in younger (<65 years) and older (≥65 years) subgroups; however, in women, no significant difference was found in these values (Additional file 1 and Additional file 2).

**Figure 3:**
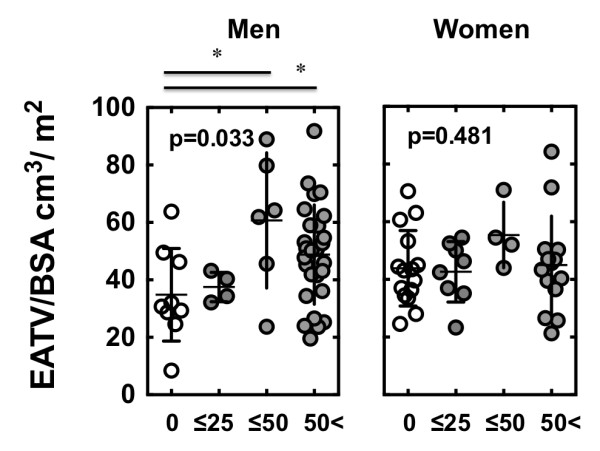
**EATV/BSA in subjects with degrees of coronary luminal stenosis.** Subjects were segregated into the following categories based on the degree of coronary luminal stenosis determined using MDCT: grade 0 = no plaque in the major coronary arteries; grade 1 = ≤25% luminal narrowing; grade 2 = ≤50% luminal narrowing; grade 3 = >50% luminal narrowing. One-way ANOVA was performed followed by Tukey-Kramer HSD test. *p < 0.05 vs. grade 0.

**Figure 4:**
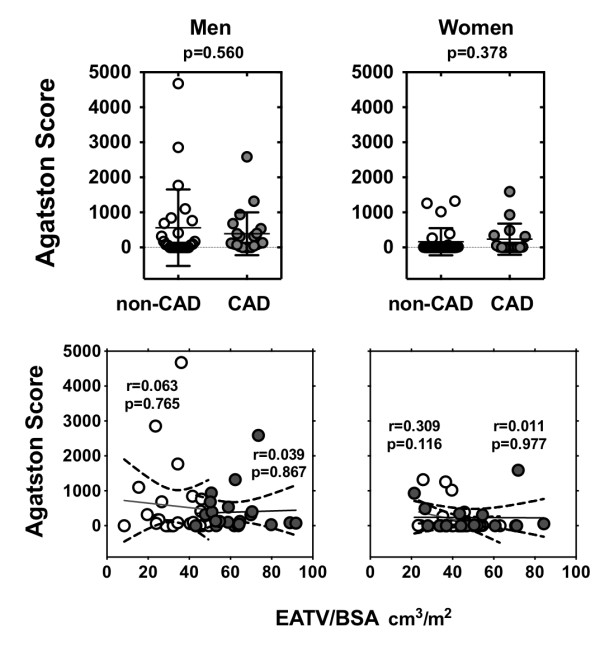
**Comparison of Agatston score in Non-CAD (○) and CAD (**●**) subjects and linear correlation between EATV/BSA and Agatston score in men and women.** EATV, epicardial adipose tissue volume; BSA, body surface area. Coronary artery disease (CAD) was defined if one has plaque lesion(s) resulting in >50% luminal narrowing. Linear correlation between EATV/BSA and Agatston score in men and women.

Next, we examined the relationship between the degree of coronary artery stenosis and EATV. Subjects were divided into the following categories: grade 0 = no plaques in the major coronary branches; grade 1 = ≤25% luminal narrowing; grade 2 = ≤50% luminal narrowing; grade 3 = >50% luminal narrowing. EATV/BSA was larger among men in grade 2 and grade 3 than in grade 1 (Figure 3). In men and women, the Agatston score did not differ between the non-CAD and CAD groups (Figure 4, upper panel). There was no correlation between EATV/BSA and Agatston score in the non-CAD and CAD groups (Figure 4, lower panel).

### Correlation between EATV/BSA and variables

**Figure 5:**
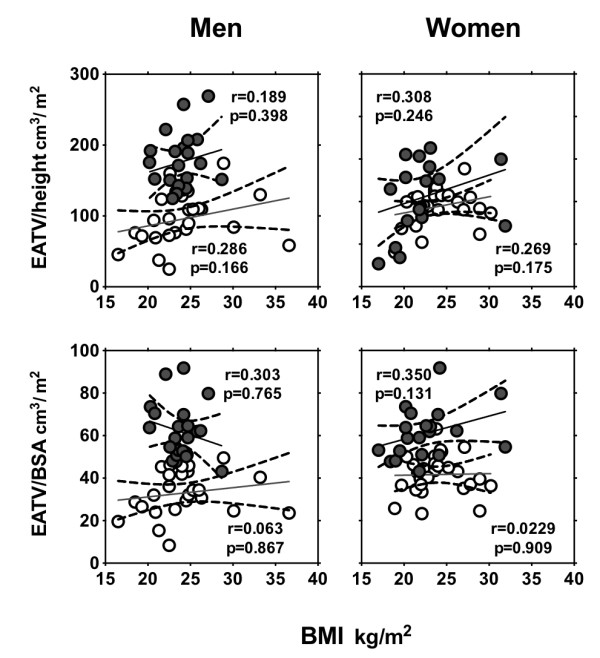
**Linear correlation between BMI and EATV/height (upper panel) and EATV/BSA (lower panel) in men and women.** Lines were plotted in non-CAD (○) and CAD subjects (●). EATV, epicardial adipose tissue volume; BSA, body surface area. Coronary artery disease (CAD) was defined if one has plaque lesion(s) resulting in >50% luminal narrowing.

**Figure 6:**
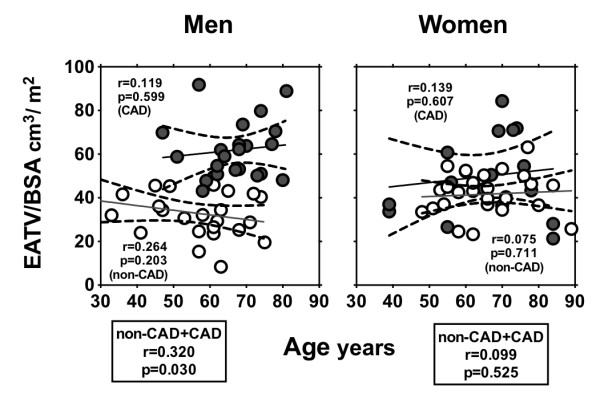
**Linear correlation between age and EATV/BSA in men and women.** Lines were plotted in Non-CAD (○) and CAD subjects (●), respectively. EATV, epicardial adipose tissue volume; BSA, body surface area. Coronary artery disease (CAD) was defined if one has plaque lesion(s) resulting in >50% luminal narrowing.

When non-CAD and CAD groups were combined, EATV, EATV/height, and EATV/BSA were not correlated with BMI (data not shown). Even after segregation into non-CAD and CAD groups, EATV/height and EATV/BSA were not correlated with BMI (Figure 5). When non-CAD and CAD groups were combined, EATV/BSA correlated with age in men (r = 0.320; p = 0.032), and the correlation was lost when men were segregated into non-CAD and CAD groups (Figure 6). There was no correlation between EATV/BSA and age in women (Figure 6). EATV/BSA correlated with the presence of diabetes mellitus in women (r = 0.431; p = 0.009) but not in men (r = 0.029; p = 0.657). EATV/BSA was not correlated with the presence of hypertension and hyperlipidemia in either men or women.

### Predictors for coronary atherosclerotic lesions

By univariate logistic regression analysis, presence of coronary atherosclerotic lesions, defined by >50% luminal narrowing, correlated with EATV, EATV/BSA, EATV/height, and age in men (Table 2). On the other hand, the presence of coronary atherosclerotic lesions correlated with EATV and EATV/BSA in women (Table 2). Body weight, BMI, systolic and diastolic blood pressure, HbA_1c_, triglycerides, LDL- and HDL-cholesterol, and the presence of hypertension and diabetes mellitus were not associated with the presence of coronary atherosclerotic lesions. Multivariate logistic regression analysis indicated that EATV/BSA was detected as an independent risk factor for >50% luminal narrowing only in men (Table 3). BMI, age, presence of hypertension, diabetes mellitus, and hyperlipidemia were not associated with the presence of coronary atherosclerotic lesions.

**Table 2:** Univariate analysis to estimate coronary atherosclerotic lesions in men and women

	**Men**			**Women**		
	**estimate**	**SE**	**P**	**estimate**	**SE**	**P**
EATV (cm^3^)	0.012	0.002	<.0001	0.009	0.004	0.023
EATV/height (cm^3^/m^2^)	0.007	0.001	<.0001	0.003	0.002	0.269
EATV/BSA (cm^3^/m^2^)	0.021	0.003	<.0001	0.015	0.006	0.015
Body weight (kg)	-0.002	0.007	0.737	-0.006	0.010	0.538
BMI (kg/m^2^)	-0.008	0.022	0.730	-0.035	0.024	0.151
Age (years)	0.017	0.005	0.003	-0.009	0.007	0.189
Systolic blood pressure (mmHg)	-0.005	0.005	0.339	0.002	0.004	0.534
Diastolic blood pressure (mmHg)	0.006	0.010	0.524	-0.011	0.010	0.319
Hypertension (yes/no)	0.224	0.164	0.181	0.057	0.145	0.698
HbA_1c_ (NGSP %)	-0.001	0.069	0.988	-0.006	0.118	0.963
Diabetes mellitus (yes/no)	0.148	0.154	0.340	0.252	0.191	0.197
LDL-cholesterol (mmol/L)	0.004	0.006	0.545	-0.005	0.008	0.570
HDL-cholesterol (mmol/L)	-0.014	0.008	0.088	0.003	0.006	0.611
Triglycerides (mmol/L)	0.000	0.001	0.642	0.000	0.001	0.833
Hyperlipidemia (yes/no)	0.200	0.228	0.386	-0.488	0.187	0.018

*SE*: standard error, *CAD*: coronary artery disease; *LDL*: low density lipoprotein; *HDL*: High density lipoprotein; *HbA*_*1c*_ : Glycosylated hemoglobin, *NGSP*: National Glycohemoglobin Standardization Program, *EATV*: epicardial adipose tissue volume, *BSA*: body surface area.

**Table 3:** Multivariate analysis to estimate coronary atherosclerotic lesions in men and women

	**Men**				**Women**			
**R**^**2**^	**0.644**				**0.555**			
**Corrected R**^**2**^	**0.571**				**0.333**			
**P value**	**<0.0001**				**0.084**			
	**estimate**	**SE**	**t**	**P value**	**estimate**	**SE**	**t**	**P value**
Intercept	-1.257	0.603	-2.09	0.046	0.995	1.560	0.64	0.536
EATV/BSA (cm^3^/m^2^)	0.021	0.004	5.52	<.0001	0.015	0.008	1.84	0.090
BMI (kg/m^2^)	0.004	0.018	0.25	0.805	-0.026	0.032	-0.8	0.440
Age (years)	0.010	0.006	1.83	0.078	-0.009	0.012	-0.74	0.476
Hypertension (yes/no)	-0.044	0.154	-0.29	0.777	-0.088	0.195	-0.45	0.661
Diabetes mellitus (yes/no)	0.040	0.117	0.34	0.736	0.147	0.261	0.56	0.584
Hyperlipidemia (yes/no)	0.012	0.167	0.07	0.945	-0.305	0.199	-1.53	0.151

*SE*: standard error, *EATV*: epicardial adipose tissue volume; *BMI*:Body mass index; *BSA*: body surface area.

## Discussion

In the present study, we employed a method for assessing total EATV by using 256-slice MDCT and found that EATV/BSA was comparable between men and women. We discovered that EATV/BSA was correlated with the presence of coronary atherosclerosis only in men. To our knowledge, this is the first study to report gender disparity in EATV and that EATV is a determinant for coronary atherosclerosis only in men.

### Volumetric quantification of EAT

EAT is distributed asymmetrically around the heart and located mainly at the atrioventricular and interventricular grooves, around major coronary arteries and the free wall of the right ventricle, and at the apex of the left ventricle. In this study, we determined the volume of EAT by using a volumetric measurement [18-22] and sought a precise estimate of the EAT volume with an increased slice range of 300–320 (0.625-mm/slice thickness). The superior border for EATV measurements was the lower surface of the left pulmonary artery origin, while the inferior border was the left ventricular apex. All epicardial fat surrounding the proximal, middle, and distal segment of major coronary arteries was included in the volumetric measurement. It is assumed that our measure employed reliable volumetric quantification of total EAT volume.

### Gender and age difference in EATV

Mean EAT volume was higher in men than in women, while mean EATV/BSA did not differ significantly between them. Although EATV is used as the sole marker of epicardial adiposity in most previous studies [18,19] barring a few [7], EATV corrected by BSA can serve as a more accurate and reliable marker for assuming atherosclerotic risk among various types of constitution samples. In our study, EATV/BSA was not correlated with BMI in men and women (Figure 5), but correlated with age in men (r = 0.320; p = 0.030) (Figure 6) and with the presence of diabetes mellitus in women (r = 0.431; p = 0.009). EATV/BSA was not correlated with the presence of hypertension nor hyperlipidemia in men and women. The above data suggests that EATV is determined by different factors in men and women.

### Association of EATV and Coronary Atherosclerosis

Several studies have already shown that total EAT volume measured by CT is associated with the presence of coronary atherosclerosis [20-22]. Although the comparison of EAT volume in men and women has been performed previously [24], no studies have reported the gender-differentiated impact of EAT volume on coronary atherosclerosis. We found that EATV, EATV/height, and EATV/BSA were clearly higher in CAD than in non-CAD group in men (Table 1, Figure 2). Higher EATV, EATV/height, and EATV/BSA were observed both in younger (<65 years) and older (≥65 years) CAD groups in men (Additional file 1 and Additional file 2); however, in women, these values did not differ significantly between non-CAD and CAD groups. Multivariate regression analysis indicated that EATV/BSA was strongly correlated with the presence of atherosclerotic lesions in men. These findings suggest that EAT deposition may be more strongly involved than total body fat compartments in coronary atherogenesis, and that epicardial fat deposition plays a role mainly in men.

### Potential mechanisms

Our study showed that EAT volume was the most strong predictor for detecting coronary atherosclerosis. In other words, EAT could be more closely linked to the development and pathogenesis of CAD than other traditional risk factors such as hypertension, dyslipidemia, obesity, and diabetes mellitus. Currently, the mechanism by which EAT volume is associated with coronary atherosclerosis only in men is unclear. Three possible mechanisms are discussed.

#### Gender differences in EAT adipocytokine

Numerous studies suggest that the role of coronary risk factors in the progression of coronary atherosclerosis differ in men and women [25-27]. EAT volume is reported to be associated with visceral adipose tissue (VAT) volume but not with subcutaneous adipose tissue volume [2,3]. Greater VAT volume is more prevalent in men and is strongly associated with metabolic syndromes [13,14,27,28], suggesting that the gender differences in this syndrome may contribute to gender differences in CAD [19]. Park et al. [29] reported that echocardiographically determined EAT thickness, a surrogate marker of total EAT volume [30,31], was significantly increased in patients with metabolic syndrome and CAD, but the power of EAT thickness to predict metabolic syndrome and CAD was stronger in patients with less BMI (<27 kg/m^2^). Associations between whole body fat distribution and EAT volume, in men and women, need to be clarified in future studies. EAT volume can be a mere marker of metabolic syndrome or a local stimulator of atherosclerotic lesions [1-4,12,32]. Although EATV accounts for ~1% of the total body fat mass, EAT volume accounts for 15–20% of the total cardiac volume and covers ~80% of the total cardiac surface [2-4]. Products secreted by EAT can be delivered to atherosclerotic plaques via the vasa vasorum [12,32]. It has been reported that the expression of pro- and anti-inflammatory cytokines was attenuated in EAT near coronary atherosclerotic lesions [10,11,33,34]. Zhou et al. reported that decreased adiponectin mRNA expression was associated with enhanced expression of cytokines IL-6, TNF-α, or TLR4 in EAT [33]. Gao et al. reported that mRNA and protein expression of chemerin, a novel adipocytokine regulating immune responses and glucose and lipid metabolism, are higher in EAT of Chinese patients with CAD [34]. They also showed that the severity of coronary atherosclerosis is positively correlated with the chemerin mRNA level in EAT rather than its circulating level [34]. We previously reported that the expression of pro-inflammatory cytokines was positively correlated, and the expression of anti-inflammatory cytokines was negatively correlated, with the ratio of M_1_/M_2_ macrophages in epicardial adipose tissue of CAD patients [10,11]. Thus, locally produced adipocytokine could enhance the progression of coronary atherosclerosis in men.

#### Gender differences in coronary plaque burden and composition

Greater CAD event rates in men than in women could be a result of the differences in coronary artery plaque burden and composition [35,36]. The coronary plaque burden is lower in women, and plaque morphology differs by age and gender [35]. Plaque rupture with a large necrotic core and disrupted fibrous cap infiltrated by macrophage and lymphocytes is common in men and older women, while plaque erosion with no fibrous cap but with intima consist of smooth muscle and proteoglycan is common in younger women. Women have relatively less calcified plaques and mixed plaques than men [36]. Taken together, coronary plaque lesions can be more strongly activated in men through inflammatory process which is enhanced in EAT with or without the underlying metabolic syndrome [10,11]. Because no correlation was found between EATV/BSA and Agatston score in non-CAD and CAD groups (Figure 4, lower panel), gender difference in coronary calcified lesion may not be related to the impact of EATV/BSA on coronary atherosclerosis in our subjects.

#### Gender differences in coronary microvascular function

Angina-like chest pain with angiographically normal coronary arteries affects women more frequently than men [37]. These individuals often show microvascular dysfunction as detected by reduced coronary blood flow reserve (CFR) [38]. In women with microvascular dysfunction, multivariate regression analysis revealed epicardial fat thickness as the only independent predictor of microvascular dysfunction (p < 0.0001), although traditional atherosclerotic risk factors such as age, hypertension, HOMA-IR, and menopause did not predict women with abnormal microvascular function [39]. The impact of EATV on coronary microvascular dysfunction in men and women should also be studied in future studies.

### Limitations

The major limitation of this study is that the results are not conclusive; The number of patients was very small to derive definite conclusions, especially when the study population is divided in subgroups. The number of female subjects in particular was very small. There is no power analysis. Furthermore, some of the results of the study were not as expected, i.e., the absence of association of traditional cardiovascular risk factors with coronary artery disease. It appears that more subjects should be included in order to achieve adequate statistical power.

### Conclusions

By assessing EAT volume using 256-slice MDCT, we found that EATV/BSA was comparable in men and women, but EATV/BSA was an independent risk factor of significant coronary atherosclerosis in men. Gender disparity in EAT volume for detection of coronary atherosclerosis prompts us to evaluate new diagnostic strategy and the underlying mechanisms.

## Abbreviations

EAT: Epicardial adipose tissue; EATV: Epicardial adipose tissue volume; MDCT: Multi-detector computed tomography; CAD: Coronary artery disease; LDL: Low-density lipoprotein; HDL: High-density lipoprotein; BMI: Body mass index; BSA: Body surface area; HbA_1c_: Glycosylated hemoglobin; ROI: Region of interest; CTDIV: Computed tomography dose index volume; MPR: Multiplanar reconstruction; MIP: Maximum intensity projection; VR: Volume rendered; ANOVA: Analysis of variance; HSD: Honestly significant difference.

## Competing interests

The authors declare that they have no competing interests.

## Authors' contributions

DM and M Shimabukuro designed and conducted this study and drafted the manuscript; JU, ST, and MH assembled the application for multidetector computed tomography; TN contributed to patient management; DM, DF, YH, HK, TS, TI, KK, TN, KY, YT, SY, NT, HY, and TW measured EATV and participated in the analysis; TK and M Sata supervised the study. All authors read and approved the final manuscript.

## Supplementary Material

Additional file 1 Characteristics of the study population after divided to <65 years or to ≥65 years. Click here for file

Additional file 2 **Comparison of BMI (upper panel) and EAT/BSA (lower panel) in Non-CAD (○) and CAD (●)subjects with less or greater than 65 years of age.** BMI, body mass index; EATV, epicardial adipose tissue volume; BSA, body surface area. Coronary artery disease (CAD) was defined if one has plaque lesion(s) causing greater than 50% luminal narrowing. Unpaired *t* test was made between Non-CAD and CAD subjects. p: P values. Click here for file
