# Supplementary material for: Gender disparities in the association between epicardial adipose tissue volume and coronary atherosclerosis: A 3-dimensional cardiac computed tomography imaging study in Japanese subjects
Source: Cardiovasc Diabetol. 2012 Sep 10;11:106. doi: 10.1186/1475-2840-11-106 (PMC3489699; doi:10.1186/1475-2840-11-106)
Supplement: Additional file 1 — Characteristics of the study population after divided to <65 years or to ≥65 years. [file 1475-2840-11-106-S1.pdf]

Supplement 1. Characteristics of the study population after divided to <65 years or to ≥65 years

	Men						Women					
	<65 years			≥65 years			<65 years			≥65 years		
	non-CAD	CAD	P	non-CAD	CAD	P	non-CAD	CAD	P	non-CAD	CAD	P
Patient, n	20	9		5	13		13	7		14	9	
Age (years)	52 ± 12	58 ± 6	ns	72 ± 3	73 ± 5	ns	57 ± 5	53 ± 10	ns	74 ± 7	75 ± 6	ns
Body weight (kg)	69.7 ± 10.7	69.0 ± 12.4	ns	58.4 ± 3.1	62.3 ± 2.1	ns	59.8 ± 6.5	54.3 ± 9.2	ns	50.8 ± 7.9	49.0 ± 9.6	ns
Body mass index (kg/m^2)	25.0 ± 4.4	24.6 ± 2.2	ns	21.0 ± 2.8	23.3 ± 1.8	ns	24.8 ± 2.4	22.3 ± 4.3	0.039	22.9 ± 3.2	22.2 ± 4.2	ns
Systolic blood pressure (mmHg)	134 ± 18	133 ± 16	ns	142 ± 11	130 ± 8	0.028	128 ± 17	118 ± 8.5	ns	135 ± 10	144 ± 23.8	ns
Diastolic blood pressure (mmHg)	75 ± 8	75 ± 11	ns	77 ± 4.6	78 ± 7.1	ns	77 ±7	72 ± 4	ns	77 ± 5	80 ± 5.0	ns
LDL-cholesterol (mmol/L)	2.47 ± 0.46	2.72 ± 0.57	ns	2.48 ± 0.33	2.52 ± 0.61	ns	3.41 ± 0.25	2.91 ± 0.90	ns	2.78 ± 0.48	2.79 ± 0.73	ns
HDL-cholesterol (mmol/L)	1.19 ± 0.39	0.96 ± 0.15	ns	1.07 ± 0.18	0.99 ± 0.18	ns	1.90 ± 0.48	1.06 ± 0.04	ns	1.52 ± 0.54	1.62 ± 0.60	ns
Triglyceride (mmol/L)	1.46 ± 0.55	1.76 ± 0.89	ns	1.04 ± 0.51	1.78 ± 0.48	ns	1.57 ± 0.14	1.78 ± 0.31	ns	1.24 ± 0.67	1.24 ± 0.66	ns
HbA_{1c} (NGSP %)	6.03 ± 0.32	7.47 ± 2.38	ns	5.98 ± 0.70	7.32 ± 1.63	ns	5.87 ± 0.21	-	ns	6.61 ± 1.26	5.75 ± 0.67	ns
Hypertension (%)	61	75	ns	67	85	ns	55	0	ns	60	75	ns
Diabetes mellitus (%)	28	50	ns	50	46	ns	0	50	ns	20	25	ns
Hyperlipidemia (%)	85	83	ns	60	92	ns	80	50	ns	78	0	ns
EATV (cm^3)	61 ± 20	109 ± 27	<0.0001	43 ± 21	104 ± 20	<0.001	64 ± 15	64 ± 24	ns	60 ± 13	72 ± 33	ns
EATV/height (cm^3/m^2)	65 ± 16	37 ± 12	<0.0001	26 ± 12	64 ± 12	<0.0001	41 ± 17	41 ± 19	ns	41 ± 9	47 ± 20	ns
EATV/BSA (cm^3/m^2)	34 ± 10	61 ± 16	<0.0001	28 ± 12	62 ± 11	<0.001	41 ± 10	43 ± 11	ns	42 ± 9	55 ± 21	ns

Values are means ± SD. P values vs Non-CAD. ns: not significant. CAD: coronary artery disease; LDL: low density lipoprotein; HDL: High density lipoprotein; HbA_{1C} : Glycosylated hemoglobin, NGSP: National Glycohemoglobin Standardization Program, EATV: epicardial adipose tissue volume, BSA: body surface area.
