# Supplementary figures and images for: Gender disparities in the association between epicardial adipose tissue volume and coronary atherosclerosis: A 3-dimensional cardiac computed tomography imaging study in Japanese subjects
Source: Cardiovasc Diabetol. 2012 Sep 10;11:106. doi: 10.1186/1475-2840-11-106 (PMC3489699; doi:10.1186/1475-2840-11-106)

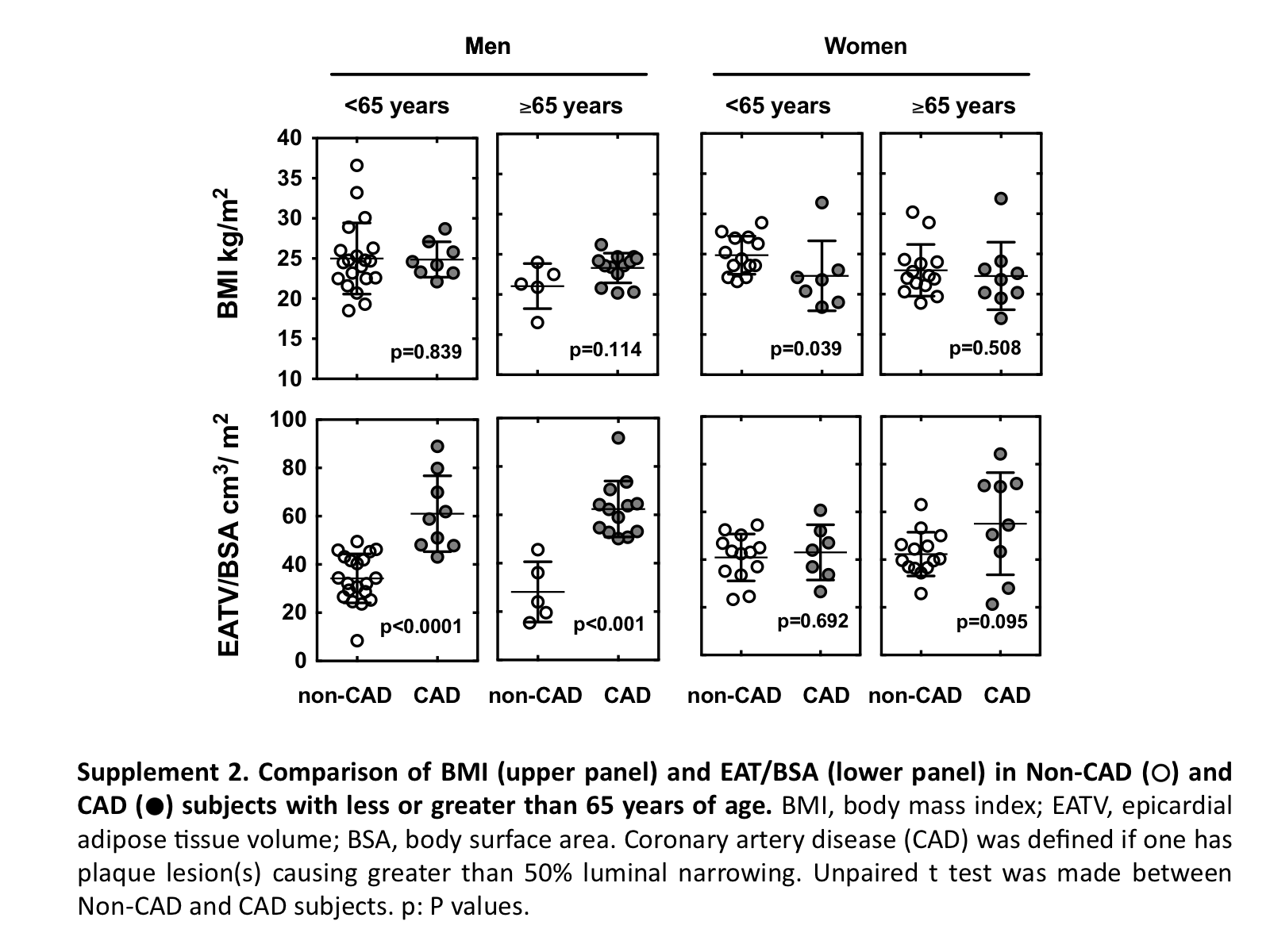

Supplement: Additional file 2 — Comparison of BMI (upper panel) and EAT/BSA (lower panel) in Non-CAD (○) and CAD (●)subjects with less or greater than 65 years of age. BMI, body mass index; EATV, epicardial adipose tissue volume; BSA, body surface area. Coronary artery disease (CAD) was defined if one has plaque lesion(s) causing greater than 50% luminal narrowing. Unpaired t test was made between Non-CAD and CAD subjects. p: P values. [file 1475-2840-11-106-S2.tiff]
